# Biochemical characterization of a *Neisseria meningitidis* polysialyltransferase reveals novel functional motifs in bacterial sialyltransferases

**DOI:** 10.1111/j.1365-2958.2007.05862.x

**Published:** 2007-09

**Authors:** Friedrich Freiberger, Heike Claus, Almut Günzel, Imke Oltmann-Norden, Justine Vionnet, Martina Mühlenhoff, Ulrich Vogel, Willie F Vann, Rita Gerardy-Schahn, Katharina Stummeyer

**Affiliations:** 1 Abteilung Zelluläre Chemie, Medizinische Hochschule Hannover Carl-Neuberg-Str. 1, 30625 Hannover, Germany; 2 Institute for Hygiene and Microbiology, University of Würzburg Josef-Schneider-Str. 2, 97080 Würzburg, Germany; 3 Laboratory of Bacterial Toxins, Center for Biologics Evaluation and Research US FDA, 8800 Rockville Pike, Bethesda, MD 20892, USA

## Abstract

The extracellular polysaccharide capsule is an essential virulence factor of *Neisseria meningitidis*, a leading cause of severe bacterial meningitis and sepsis. Serogroup B strains, the primary disease causing isolates in Europe and America, are encapsulated in α-2,8 polysialic acid (polySia). The capsular polymer is synthesized from activated sialic acid by action of a membrane-associated polysialyltransferase (*Nm*B-polyST). Here we present a comprehensive characterization of *Nm*B-polyST. Different from earlier studies, we show that membrane association is not essential for enzyme functionality. Recombinant *Nm*B-polyST was expressed, purified and shown to synthesize long polySia chains in a non-processive manner *in vitro*. Subsequent structure–function analyses of *Nm*B-polyST based on refined sequence alignments allowed the identification of two functional motifs in bacterial sialyltransferases. Both (D/E-D/E-G and HP motif) are highly conserved among different sialyltransferase families with otherwise little or no sequence identity. Their functional importance for enzyme catalysis and CMP-Neu5Ac binding was demonstrated by mutational analysis of *Nm*B-polyST and is emphasized by structural data available for the *Pasteurella multocida* sialyltransferase *Pm*ST1. Together our data are the first description of conserved functional elements in the highly diverse families of bacterial (poly)sialyltransferases and thus provide an advanced basis for understanding structure–function relations and for phylogenetic sorting of these important enzymes.

## Introduction

*Neisseria meningitidis* (*Nm*) is a leading cause of bacterial meningitis and sepsis in children and adolescents. Sporadic cases as well as outbreaks and epidemic waves are observed. Despite the availability of potent antimicrobial agents, case-fatality rates are high and survivors frequently suffer from sequelae such as limb loss and deafness (van Deuren *et al*., 2000). Essential virulence factors of disease causing meningococci are their extracellular polysaccharide capsules. Serogroup B strains (*Nm*B), the primary disease-causing isolates in Europe and America, are encapsulated in α-2,8 polysialic acid (polySia). The *Nm*B capsule was shown to mediate resistance to phagocytosis and complement-mediated bacteriolysis and it is chemically and immunologically identical to polySia found in the human host (Mackinnon *et al*., 1993; Hammerschmidt *et al*., 1994; Vogel *et al*., 1997; Vogel and Frosch, 1999). This mimicry also prevents the generation of effective polysaccharide-based vaccines against *Nm*B strains. Bypass of host defence mechanisms by polySia capsules has not only been described for *Nm*B, but is also an important virulence determinant of other disease-causing pathogens such as *Escherichia coli* K1 and K92, *Moraxella nonliquefaciens*, *Pasteurella haemolytica* and *N. meningitidis* serogroup C (Troy, 1992). The polySia capsules of *Nm*C and *E. coli* K92 are connected by α-2,9 (Bhattacharjee *et al*., 1975) or alternating α-2,8/α-2,9 (Egan *et al*., 1977) glycosidically linked sialic acids, respectively. Because of their critical function in bacterial pathogenesis enzymes involved in polySia biosynthesis are interesting targets for therapeutic intervention. Biosynthesis of polySia is catalysed by membrane-associated polysialyltransferases (polySTs) at the cytoplasmic side of the inner membrane of Gram-negative bacteria (Masson and Holbein, 1983; Troy, 1992). The polymerization reaction proceeds by transfer of sialic acid from the donor substrate CMP-Neu5Ac to the non-reducing end of a growing polySia chain and was proposed to be processive, as no reaction intermediates could be detected (Steenbergen and Vimr, 2003). Polysialyltransferases have been cloned from *E. coli* K1 and K92 as well as from *N. meningitidis* serogroup B and C (Weisgerber *et al*., 1991; Vimr *et al*., 1992; Edwards *et al*., 1994; Claus *et al*., 1997). Identity is highest between the pair of *E. coli* (82% identity) and meningococcal enzymes (65% identity) and is lower between the two genera (33% identity). Interestingly, the neisserial enzymes are elongated by a C-terminal domain not present in the *E. coli* polySTs.

While only few studies included the neisserial polySTs (Masson and Holbein, 1983; Swartley *et al*., 1997; Steenbergen and Vimr, 2003), the *E. coli* enzymes have been studied more extensively (Whitfield, 2006; Troy, 1992). They are unable to start *de novo* biosynthesis of polySia, but elongate exogenously added acceptors including oligo- and polysialic acids, sialylgangliosides and synthetic acceptors (Steenbergen and Vimr, 1990; Cho and Troy, 1994; McGowen *et al*., 2001). The nature of the priming endogenous acceptor is still unknown, but recently gene products NeuE and KpsC were shown to be required for *de novo* synthesis of polySia in *E. coli* (Andreishcheva and Vann, 2006) and the functional complex of the *E. coli* K92 polysialyltransferase was found to be larger than a monomer (Vionnet *et al*., 2006). Construction of chimeric proteins between the closely related *E. coli* K1 and K92 enzymes, synthesizing α-2,8- and alternating α-2,8/α-2,9-linked polySia, respectively, mapped the region responsible for linkage specificity to primary sequence elements located between amino acids 53 and 85 in both enzymes (Steenbergen and Vimr, 2003). However, so far no data on isolated proteins are available. Attempts to purify or solubilize polySTs failed and resulted in inactivation of the enzymes and it was therefore proposed that membrane association is required for polyST activity (Steenbergen and Vimr, 2003; Vionnet *et al*., 2006). The lack of purified native or recombinant protein furthermore prevented detailed structure function analyses of these important enzymes.

No conserved motifs have been described for bacterial sialyltransferases. This is different from the eukaryotic sialyltransferases, where four conserved motifs have been shown to be involved in binding of donor and acceptor substrates and in enzyme catalysis (Drickamer, 1993; Livingston and Paulson, 1993; Geremia *et al*., 1997; Jeanneau *et al*., 2004). Moreover, based on primary sequence similarities all eukaryotic sialyl- and polysialyltransferases can be grouped into a single family GT-29 of the CAZy database (http://www.cazy.org) while the bacterial enzymes are distributed into four families (Coutinho *et al*., 2003). Bacterial polysialyltransferases are found in CAZy family GT-38, while families GT-42, GT-52 and GT-80 contain sialyltransferases that sialylate bacterial lipooligosaccharide (LOS). Structural information is available for the *Campylobacter jejuni* sialyltransferase cst-II (member of CAZy family GT-42) (Chiu *et al*., 2004) and the sialyltransferase *Pm*ST1 of *Pasteurella multocida* (member of CAZy family GT-80) (Ni *et al.*, 2006). While cst-II belongs to the glycosyltransferase-A-like (GT-A) structural group, *Pm*ST1 has a GT-B-like fold.

In the current study we focused on the characterization of the polyST from *N. meningitidis* serogroup B and throughout succeeded with soluble expression and purification of recombinant *Nm*B-polyST. We thereby demonstrate that membrane association is not a prerequisite for the formation of functional enzyme. We also show that removal of the C-terminal extension present in *Nm*B but not in the homologous *E. coli* enzymes, completely abolished enzymatic activity, proving it as an essential functional domain. Using site-directed mutagenesis and refined protein alignment strategies, we identified two functionally important motifs, which are highly conserved in a number of bacterial (poly)sialyltransferases of otherwise unrelated sequences. With these data we provide the first evidence for the conservation of catalytic features among bacterial sialyl- and polysialyltransferases and thereby improve the basis for design of sialyltransferase-specific drugs.

## Results

### Recombinant expression of *Nm*B-polyST

**Fig. 1:**
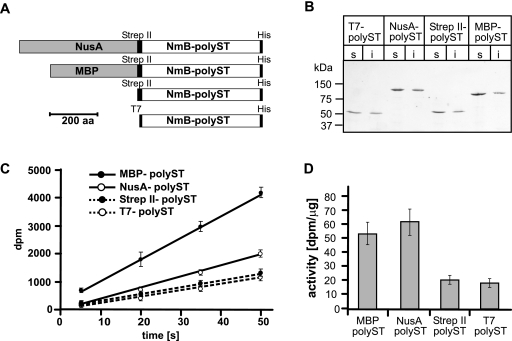
Influence of N-terminal fusion tags on *NmB*-polyST expression and activity. A. Schematic representation of *Nm*B-polyST fusion proteins. *Nm*B-polyST is shown as white box while short epitope tags (His, T7, Strep II) and large fusion partners (MBP, NusA) are given as black and grey boxes respectively. The length of the black ruler represents 200 amino acids. B. Western blot analysis of *Nm*B-polyST fusion proteins. Proteins were expressed in *E. coli* BL21(DE3) and equal amounts of soluble (s) and insoluble (i) fractions were separated by SDS-PAGE. C-terminally epitope-tagged fusion proteins were detected by Western blot analysis with anti-His-tag antibody. C. Enzymatic activity of *Nm*B-polyST fusion proteins in the soluble fractions. PolyST activity was analysed using the radiochemical activity assay. Reactions were incubated at room temperature and aliquots were assayed for radiolabelled polySia at the indicated time points. Each value represents the average of three independent determinations with the standard deviation indicated. D. Specific activities of *Nm*B-polyST fusion proteins were standardized by *Nm*B-polyST expression levels, which were determined by immunoblotting and infrared fluorescence detection.

Detailed structure–function analyses of bacterial polysialyltransferases were hitherto prevented by insufficient supply of the enzymes. Expression levels were described to be low and polyST activity was found associated with bacterial membranes (Shen *et al*., 1999; Steenbergen and Vimr, 2003; Vionnet *et al*., 2006). As attempts to solubilize polyST were either unsuccessful or accompanied by inactivation of the enzyme, all analyses have been performed with crude membrane fractions as enzyme source. With the aim of obtaining purified enzyme in yields sufficient to perform structure–function studies, we began the current work with a systematic search for conditions that would allow production of recombinant *Nm*B-polyST. To test the influence of N-terminal fusion partners on *Nm*B-polyST expression and activity, constructs were generated either with short N-terminal epitope tags (T7, Strep II) or with additional large fusion parts like NusA and maltose-binding protein (MBP) (Fig. 1A). The constructs were expressed in *E. coli* BL21 (DE3) and soluble and insoluble fractions of the bacterial lysates were analysed for expression and activity of *Nm*B-polyST. Although soluble protein could be detected for all constructs (Fig. 1B), the addition of large N-terminal fusions considerably increased the amount of active protein in the soluble fractions (Fig. 1C). Compared with polySTs carrying only short N-terminal epitope tags, additional fusion of NusA or MBP increased the soluble activity of NusA– and MBP–polyST two- and threefold, respectively. Also, the specific activity of both fusion proteins was increased twofold compared with enzymes with short tags (Fig. 1D). Subsequent experiments were therefore carried out with either NusA or MBP fusion proteins.

### Definition of the minimal active domain of *Nm*B-polyST

**Fig. 2:**
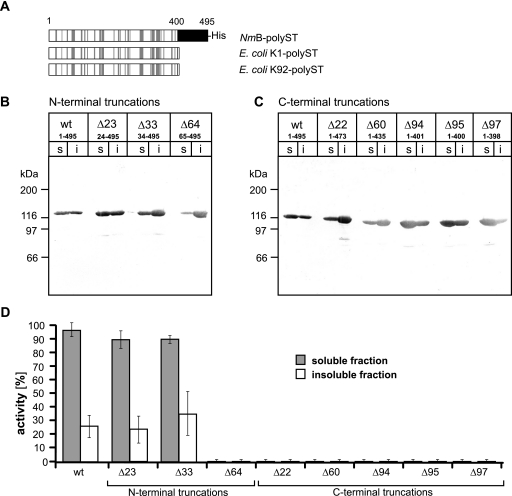
Analysis of expression and activity of wild-type and truncated *NmB*-polyST. A. Schematic representation of bacterial polySTs. The N-terminal domain homologous in polysialyltransferases of *E. coli* K1, K92 and *N. meningitidis* serogroup B is shown as white box, with conserved amino acid stretches indicated in grey. The 95 amino acid comprising C-terminal domain of *Nm*B-polyST, which is not conserved in the *E. coli* enzymes, is depicted in black. B and C. Western blot analysis of wild-type and truncated *Nm*B-polySTs. NusA fusion proteins of full-length and N-terminally (B) and C-terminally (C) truncated *Nm*B-polyST were expressed in *E. coli* BL21(DE3). Soluble (s) and insoluble (i) fractions (re-suspended in the same volume and buffer as the soluble fractions) were prepared and equal amounts were analysed by SDS-PAGE and Western blotting using anti-His-tag antibody. D. Enzymatic activity of soluble and insoluble fractions was analysed using the radiochemical polyST assay. The data were standardized by *Nm*B-polyST expression levels that were determined by immunoblotting and infrared fluorescence detection, and are given relative to the enzymatic activity of the soluble wild-type fraction. Each value represents the average of three independent determinations with the standard deviation indicated.

It has been reported for other bacterial sialyltransferases that N- or C-terminal truncations significantly increase protein solubility by eliminating membrane interaction domains (Chiu *et al*., 2004; Ni *et al*., 2006). Consequently, the second series of experiments was designed to define the minimal catalytic domain of *Nm*B-polyST. N- and C-terminal truncations of the enzyme were generated as NusA fusion proteins carrying a C-terminal His-tag for detection. Full-length and truncated proteins were expressed in *E. coli* and soluble and insoluble cell fractions were tested for expression and enzymatic activity of *Nm*B-polyST. As depicted in Fig. 2, approximately 50% of the wild-type polyST was soluble (Fig. 2B) and enzymatically active (Fig. 2D), whereby the detected activity was threefold higher in the soluble than in the insoluble fraction. Removal of 23 (Δ23*Nm*B-polyST) and 33 (Δ33*Nm*B-polyST) amino acids from the N-terminus had only slight effects on solubility and activity of *Nm*B-polyST. However, deletion of the first 64 amino acids (Δ64*Nm*B-polyST) shifted the majority of the expressed protein to the insoluble fraction and no enzymatic activity was detected in soluble or insoluble fractions.

Primary sequence analysis revealed that *Nm*B-polyST carries a C-terminal extension of 95 amino acids that is not present in the polySTs of *E. coli* K1 and *E. coli* K92 (Fig. 2A). To investigate the role of this additional protein part, we generated a set of truncated *Nm*B-polySTs lacking the C-terminal domain either partially (*Nm*B-polySTΔ22, *Nm*B-polySTΔ60) or completely (*Nm*B-polySTΔ94, *Nm*B-polySTΔ95, *Nm*B-polySTΔ97). As displayed in Fig. 2C, all variants could be expressed as soluble proteins at similar or, in the case of constructs with entirely deleted C-terminal domain, even higher levels than the full-length enzyme. However, each C-terminal truncation completely abolished enzymatic activity (Fig. 2D), indicating that the C-terminal domain is indispensable for *Nm*B-polyST activity.

### The fusion protein MBP–*Nm*B-polyST produces capsular polySia *in vivo*

**Fig. 3:**
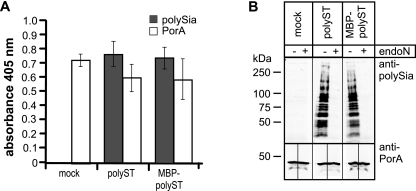
*In vivo* activity of *Nm*B-polyST fused to maltose-binding protein (MBP). A. MBP–*Nm*B-polyST and native *Nm*B-polyST carrying no additional tags were cloned into a neisserial expression vector and transformed into the polyST-deficient *Neisseria* strain 2517. PolySia capsules of parental strain (mock) and transformants were analysed by whole-cell ELISA using mab 735. Equal loading of the wells with *Neisseria* was controlled using mab P1.2 for detection of the meningococcal major outer membrane protein PorA. Each value represents the average of three independent determinations with the standard deviation indicated. B. Lysates of the parental strain (mock) and transformants were additionally analysed by SDS-PAGE and Western blotting using mab 735 before and after treatment with polySia-degrading endoN (top). Equal sample loading was confirmed in a parallel Western blot immunostained with anti-PorA antibody P1.2 (bottom).

Our efforts to express recombinant *Nm*B-polyST in the *E. coli* expression strain BL21 (DE3) clearly revealed beneficial effects of large N-terminal fusion parts on the expression of active, soluble enzyme. However, to analyse if polyST fusion proteins maintain enzymatic activity also *in vivo*, wild-type and MBP–*Nm*B-polyST were subcloned into a neisserial expression vector and transformed into the polyST-deficient neisserial strain 2517. Parental strain and transformants were analysed for capsular polySia in a quantitative whole-cell ELISA. Equal loading of the microtitre plates with bacteria was confirmed in a parallel ELISA directed against the meningococcal major outer membrane protein PorA. Interestingly, no difference in capsule expression was observed between strains complemented with wild-type or the MBP fusion construct (Fig. 3A). Moreover, Western blot analysis of the neisserial lysates revealed a similar polySia staining of wild-type and fusion protein that was not detectable in samples treated with endoN (Fig. 3B), which is a bacteriophage-derived enzyme that degrades polySia with high substrate specificity (Mühlenhoff *et al*., 2003; Stummeyer *et al*., 2005). This clearly demonstrates that MBP–*Nm*B-polyST is enzymatically active *in vivo*.

### Purification of recombinant *Nm*B-polyST

**Fig. 4:**
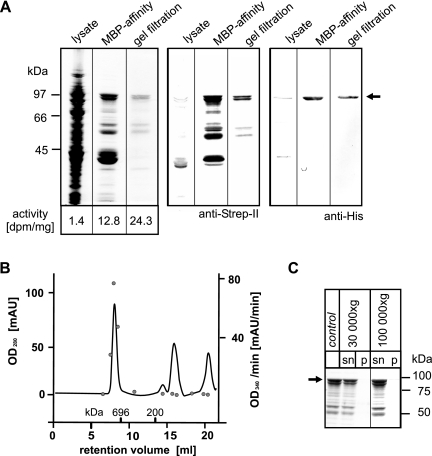
Purification of *NmB-*polyST. A. The MBP fusion protein of *Nm*B-polyST was expressed in *E. coli* BL21 (DE3) and purified in a sequence of MBP affinity and size exclusion chromatography. Protein containing fractions were analysed by Coomassie-stained SDS-PAGE (left) and Western blot directed against the N-terminal StrepII-tag (middle) and the C-terminal His-tag (right). Protein bands corresponding to MBP–*Nm*B-polyST are indicated by an arrow. Enzyme activity was monitored by the radiochemical polyST assay and specific activities were calculated as indicated. B. Size exclusion chromatography of MBP–*Nm*B-polyST. Elution positions of standard proteins [Thyroglobulin (669 kDa), β-Amylase (200 kDa)] are indicated. Enzymatic activity of the collected fractions (secondary *y*-axis, grey circles) was determined using the enzyme-linked polyST assay. C. High-speed centrifugation of purified *Nm*B-polyST. Samples were centrifuged as indicated and equal amounts of supernatant (sn) and pellet (p) fraction, which was re-suspended in the same volume as the supernatant, were analysed by Coomassie-stained SDS-PAGE.

As MBP–*Nm*B-polyST was shown to be active *in vitro* and *in vivo* and because MBP could be directly utilized for affinity chromatography, purification was optimized for the MBP–*Nm*B-polyST construct schematically depicted in Fig. 1A. The fusion construct additionally carries two short epitope tags (N-terminal Strep II-, C-terminal His-tag), which were used for detection of the protein throughout the purification. After overexpression in *E. coli* BL21(DE3) at 15°C, the recombinant MBP–*Nm*B-polyST was purified in two consecutive steps by MBP affinity and size exclusion chromatography. Trials to also utilize the C-terminal His-tag for purification failed, indicating that the epitope is not accessible in the native enzyme. As shown in Fig. 4A, the applied purification procedure yielded a highly enriched MBP–*Nm*B-polyST (protein of 100 kDa). Major bands of smaller molecular weight still visible in the Coomassie-stained SDS-PAGE of the purified pool were also detected in a Western blot directed against the StrepII-tag, but not in a blot stained with an anti-His-tag antibody. This indicates that these bands represent breakdown products of MBP–*Nm*B-polyST caused by C-terminal degradation.

Throughout the purification, polyST activity was monitored by the radiochemical polyST assay using colominic acid as the acceptor. Enzyme activity was detected in all MBP–*Nm*B-polyST-containing fractions and specific activity increased about 20-fold from cell lysate to the elute obtained after gel filtration (see Fig. 4A, bottom). As depicted in Fig. 4B, the fusion protein eluted in a single peak at the upper limit of the applied Superdex 200 gel filtration column, which suggests association of MBP–*Nm*B-polyST as hexameric or higher-order oligomers. However, the purified protein was soluble and remained completely in the supernatant after high-speed centrifugation (100 000 *g*, Fig. 4C). One litre of expression culture yielded 1.3 mg of the highly enriched MBP–*Nm*B-polyST. The protein was concentrated to 2 mg ml^−1^ and stored at 4°C for 3–4 weeks without detectable loss of activity or further degradation.

### Soluble recombinant MBP–*Nm*B-polyST produces polySia chains in a non-processive manner

**Fig. 5:**
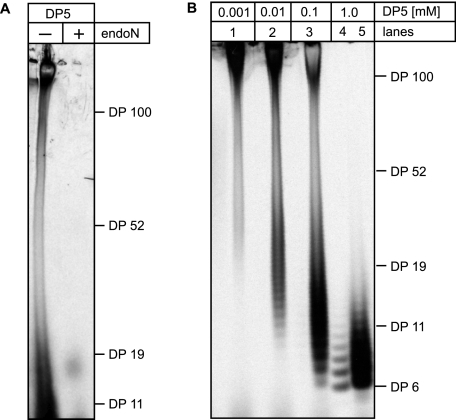
PolySia biosynthesis of purified *Nm*B-polyST. A. Purified *Nm*B-polyST was assayed for 30 min in the presence of 0.1 mM pentameric α2,8-linked sialic acid (DP5) and 1 mM CMP-[^14^C]-Neu5Ac. Subsequently, half of the sample was digested with polySia-specific endoN. Radiolabelled reaction products were separated by acrylamide gel electrophoresis (10%) and detected by autoradiography. The following dyes were used as standards and correspond to polySia chain length given in brackets: trypan blue (DP100), xylene cyanol (DP52), bromophenol blue (DP19), bromcresol purple (DP11). B. Dependence of chain length distribution on acceptor concentration. The assay was performed as described in (A) but DP5 concentrations were varied as indicated. Samples were separated on a 25% polyacrylamide gel. To display single oligomeric-reaction products at the highest acceptor concentration more clearly, less sample volume was applied in lane 4.

**Fig. 6:**
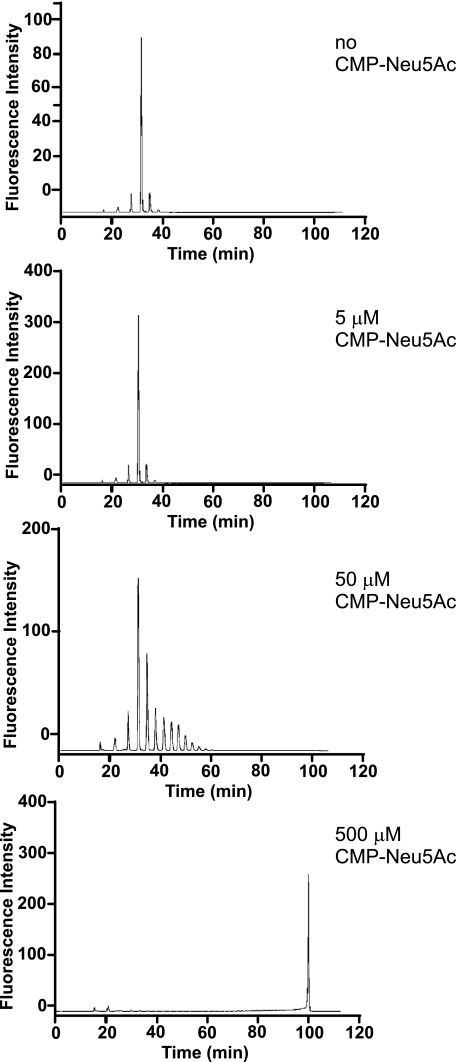
Dependence of GT3-FCHASE extension by purified *Nm*B-PST on CMP-Neu5Ac concentration. Purified *Nm*B-polyST was incubated with GT3-FCHASE and CMP-Neu5Ac for 5 min as described in *Experimental procedures*. The respective CMP-Neu5Ac concentrations are indicated. The reaction mixtures were then adjusted to 25% ethanol. The supernatants were applied to a DNA Pac PA-100 column and chromatographed with a gradient of NaNO_3_ according to Inoue *et al*. (2001) and Inoue and Inoue (2003).

**Fig. 7:**
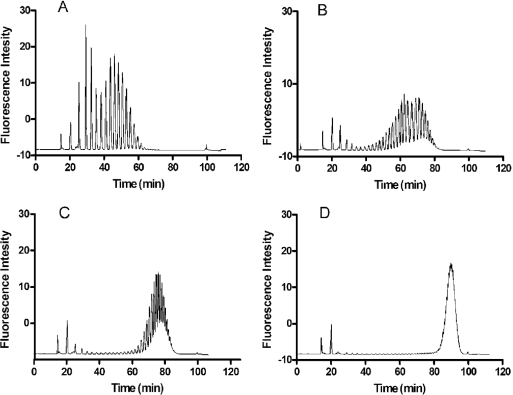
Time-course of elongation of GT3-FCHASE by *Nm*B-polyST. Purified *Nm*B-polyST (37 μg ml^−1^) was incubated with GT3-FCHASE and 0.5 mM CMP-Neu5Ac for (A) 2 min, (B) 5 min, (C) 10 min and (D) 30 min prior to adjusting the reaction mixtures to 25% ethanol. The supernatants were applied to a DNA Pac PA-100 column and chromatographed with a gradient of NaNO_3_ according to Inoue *et al*. (2001) and Inoue and Inoue (2003).

Capsular polysaccharide purified from serogroup B *N. meningitidis* was shown to exhibit a high degree of polymerization (DP) (Gotschlich *et al*., 1969; Frosch and Müller, 1993). This implies that *Nm*B-polyST is able to produce large polymers when the enzyme is part of the native capsule biosynthesis complex associated with the inner bacterial membrane. To analyse products synthesized by purified *Nm*B-polyST in more detail, pentameric (DP5) α-2,8-linked oligosialic acid was used as the primer for the recombinant enzyme and the polymerization reaction was started by addition of radiolabelled sugar donor substrate CMP-[^14^C]-Neu5Ac. Products were separated by high percentage polyacrylamide gel electrophoresis and visualized by autoradiography. Because polySia can be specifically degraded with bacteriophage endosialidases (endoN) (Mühlenhoff *et al*., 2003; Stummeyer *et al*., 2005), the nature of the synthesized products was controlled by treating one of two parallel samples with endoN prior to electrophoresis. As shown on the gel in Fig. 5A, which separates polymerization products from DP 10 to > 100, a large fraction of the synthesized polySia was longer than 100 residues per chain. All reaction products were specifically degraded by endoN. This demonstrates that the recombinant MBP–*Nm*B-polyST is able to synthesize long polySia chains starting with short oligomeric acceptors *in vitro*. However, in addition to the high-molecular-weight polySia, also shorter oligosialic acid reaction products were detectable (Fig. 5A). To validate the presence of short reaction products and analyse the mode of chain elongation in more detail, we studied the influence of acceptor concentrations on chain length distribution. Therefore, DP5 concentrations were altered over three orders of magnitude starting with 1 μM, while the concentration of enzyme and donor substrate CMP-Neu5Ac was kept constant. As shown in Fig. 5B, increasing acceptor concentrations augment the concentration of short- and medium-sized reaction products until – at the point of equimolar concentrations of DP5 and CMP-Neu5Ac (lanes 4 and 5) – virtually all radiolabelled products remain below DP11 (Fig. 5B). An identical correlation of chain length distribution and acceptor concentration was obtained for trimeric (DP3) α-2,8-linked oligo sialic acid (Fig. S1). These data argue against processivity of *Nm*B-PolyST *in vitro*, as in case of a highly processive mechanism synthesis of few long chains should be favoured over synthesis of many short chains. To evaluate these data in a second assay system, we used our recently developed fluorescence-based polyST assay (Vionnet and Vann, 2007) that utilizes the trisialylganglioside analogue GT3-FCHASE as artificial acceptor substrate. In a first experiment GT3-FCHASE was incubated with purified MBP–*Nm*B-polyST and increasing concentrations of the donor substrate CMP-Neu5Ac (0, 5, 50, 500 μM). After an incubation time of 30 min the synthesized reaction products were analysed by ion exchange HPLC. As shown in Fig. 6, the chain length of synthesized polySia increased with increasing concentrations of donor substrate and resulted in a complete conversion of the acceptor substrate to high-molecular-weight polymer at a CMP-Neu5Ac concentration of 500 μM. This clearly demonstrates synthesis of long polySia chains. To verify the suggested non-processive elongation mode of *Nm*B-polyST (Fig. 5B and Fig. S1), we performed a time-course experiment under reaction conditions that, as shown above, result in synthesis of long polySia chains (Fig. 7). If the reaction was stopped after 2 min predominantly short oligosialic acid-containing products were found (Fig. 7A) that were further elongated as the polymerization progressed (Fig. 7B and C) to finally yield high-molecular-weight polymer (Fig. 7D). This is reflected by a shift of the detected product peaks to longer retention times (Fig. 7). The occurrence of intermediates in the polymerization reaction argues, in agreement with the data obtained from the radioactive assay system, for non-processive chain elongation by MBP–*Nm*B-polyST and indicates that the enzyme dissociates from the product after each addition of sialic acid.

### Identification of two conserved motifs in bacterial sialyl- and polysialyltransferases

**Fig. 8:**
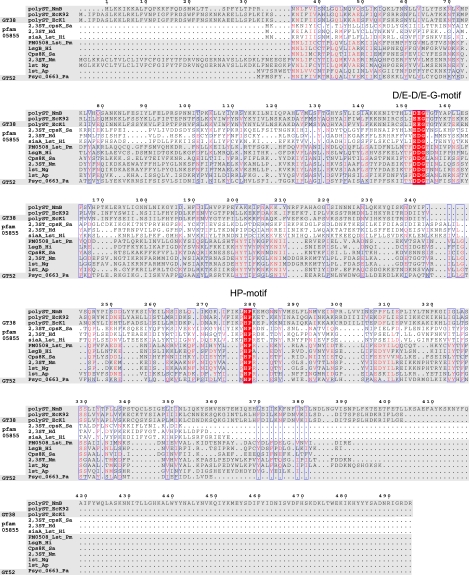
Conserved motifs in bacterial sialyl- and polysialyltransferases. Sequence alignment of bacterial sialyltransferases identifying two short motifs conserved in CAZy families GT-38 (bacterial polysialyltransferases), GT-52 (bacterial LOS-sialyltransferases) and in pfam family 05855 (similar to *H. ducreyi* sialyltransferase Lst). To improve clarity of the illustration only three representatives of pfam 05855 are shown. PolyST_*Nm*B (AAA20478), polyST_EcK92 (AAA24215), polyST_EcK1 (CAA43053), 2,3ST_cpsK_Sa (EAO062164), 2,3-ST_Hd (AAD28703), SiaA_Lst_Hi (AAL38659), PM0508_Lst_Pm (AAK02592), LsgB_Hi (AAX88755), Cps8K_Sa (AAR29926), 2,3_ST_*Nm* (AAC44544), lst_Ng (AAY41933), lst_Ap (AAS66624), Psyc_0663_Pa (AAZ18522). Alignments were made using Multalign (Corpet, 1988).

To identify amino acid residues critical for (poly)sialyltransferase activity, we searched for common sequence motifs in the available bacterial sialyltransferase sequences. By iterative steps of pairwise and multiple alignments combined with visual inspection of the sequences, we identified two short motifs (D/E-D/E-G and HP). Both motifs are present in a range of enzymes with otherwise little identity (Fig. 8) that, based on functional and sequence properties, had been allocated to different CAZy families (GT-38 and GT-52) and to pfam family 05855. While GT-38 contains bacterial polysialyltransferases, GT-52 includes bacterial LOS sialyltransferases and pfam 05855 groups sialyltransferases similar to the sialyltransferase Lst of *Haemophilus ducreyi* (Bozue *et al*., 1999). The D/E-D/E-G and the HP motif identified in this study are conserved in all members of the three families and, interestingly, are also found in the bacterial LOS sialyltransferases grouped in GT-80. However, the relation to GT-80 family members is not easily seen in an alignment, as the stretch of sequence between the D/E-D/E-G and HP motif is approximately 50 amino acids longer than in the other families.

### D/E-D/E-G and HP motifs are crucial for *Nm*B-polyST activity

**Fig. 9:**
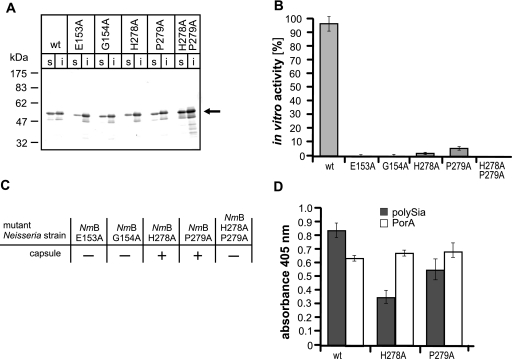
Analysis of *Nm*B-polyST mutants *in vivo* and *in vitro*. A. Single-point mutations were introduced into the D/E-D/E-G and HP motif of *Nm*B-polyST. Wild-type and mutant enzymes were expressed in *E. coli* and soluble (s) and insoluble (i) fractions of the bacterial lysates were analysed for expression by SDS-PAGE and Western blotting using anti-T7-tag antibody. B. PolyST activity was determined in protein lysates with similar expression levels by the radiochemical assay. Relative activities were calculated compared with the wild-type enzyme (100%). C. Mutant strains of *N. meningitidis* were generated by replacing the native polyST gene with mutant polySTs carrying the respective point mutations. Capsule expression was analysed with the polySia-specific antibody 735 by slide agglutination. D. Capsular polySia of mutant strains with residual capsule expression was quantified by ELISA using mab 735. Equal loading of the wells with *Nm*B was controlled using mab P1.7 for detection of the meningococcal major outer membrane protein PorA. Each value represents the average of three independent determinations with the standard deviation indicated.

To investigate the functional relevance of the identified motifs, point mutations were introduced into *Nm*B-polyST by site-directed mutagenesis. The histidine and proline of the HP motif as well as the glycine and its adjacent glutamate residue of the D/E-D/E-G motif were individually changed to alanine, resulting in the mutants H278A, P279A, E153A and G154A. Mutants were expressed in *E. coli* BL21(DE3) and lysates were analysed for protein expression and activity by Western blot and the radiochemical polyST activity assay, respectively. As shown in Fig. 9A, all mutants were expressed at the level of the wild-type protein and the ratio between soluble and insoluble protein was in all cases similar to wild type. In contrast, enzyme activity was dramatically impaired in all mutants (Fig. 9B). While mutations in the D/E-D/E-G motif (E153A and G154A) fully inactivated the protein, the H278A and P279A mutants of the HP motif maintained residual activity (below 10% of wild type). However, when both residues of the HP motif were changed to alanine simultaneously (H278A/P279A) enzyme activity was abolished.

To confirm the functional relevance of the D/E-D/E-G and HP motifs *in vivo*, mutant meningococcal strains were generated by homologous recombination and capsular phenotypes of the mutant strains were analysed by slide agglutination using the polySia-specific antibody 735. In agreement with the *in vitro* studies, *Neisseria* strains exhibiting the E153A, G154A or the double mutation H278A/P279A did not synthesize polySia capsules while capsules were still produced in strains carrying the single mutations H278A and P279A (Fig. 9C). However, a whole-cell ELISA used to quantitatively compare polySia synthesis in wild-type bacteria and mutants clearly demonstrated impairment of capsule biosynthesis in mutants with residual *in vivo* activity. While the PorA control was similar for wild type and mutants, the polySia signal was significantly reduced to 30% and 60% for the mutants carrying the amino acid exchanges H278A and P279A respectively. This demonstrates that mutations within the HP motif not only decrease enzymatic activity *in vitro*, but also reduce capsule production *in vivo*.

### An enzyme-linked assay system for the functional characterization of purified polysialyltransferases

**Fig. 10:**
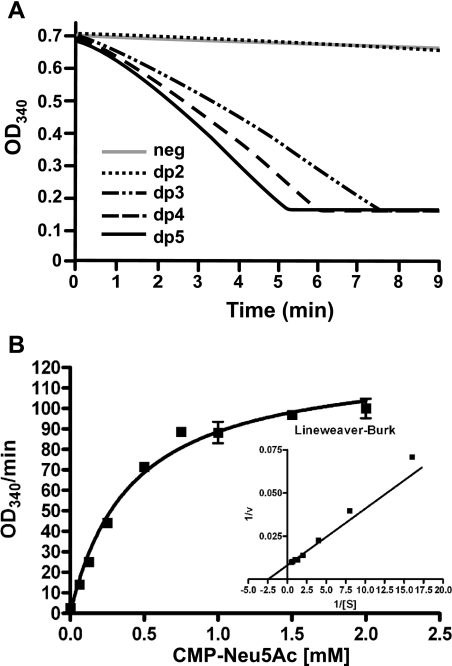
Enzymatic characterization of *Nm*B-polyST. A. To analyse acceptor specificity, oligomeric α2,8-linked sialic acids with a degree of polymerization (DP) ranging from DP2 to DP6 (0.5 mM) were assayed at constant enzyme (4 μM) and CMP-Neu5Ac concentrations (1 mM) using the continuous spectrophotometric assay. PolyST activity is detected as decreasing absorption at 340 nm (NADH oxidation). B. To determine *K*_m_ and *V*_max_ of *Nm*B-polyST for the donor substrate CMP-Neu5Ac, measurements were performed at 30°C and 0.28 mg ml^−1^ colominic acid. The resulting substrate velocity curve and Lineweaver–Burk plot are depicted. Kinetic parameters were obtained by non-linear regression in Prism (GraphPad Software).

To compare wild-type and mutant polySTs with partial activity (particularly mutants H278A and P279A) we decided to perform kinetic studies. As a first step towards this goal, the glycosyltransferase assay developed by Gosselin *et al*. (1994) was adapted to assay polyST activity. This spectrophotometric assay links the release of CMP by polyST during polySia synthesis to NADH oxidation and thus enables continuous monitoring of the enzyme reaction. PolyST activity towards short oligosialic acid acceptors (DP2 to DP5) was measured at constant CMP-Neu5Ac and enzyme concentrations. As shown in Fig. 10A, efficient chain elongation required oligomers of at least DP3. These findings are in agreement with the acceptor dependence obtained when polyST-containing membrane preparations of *E. coli* K1 or K92 were assayed in radiochemical or HPLC-based test systems (Steenbergen and Vimr, 1990; Ferrero *et al*., 1991; Chao *et al*., 1999).

Subsequently, kinetic parameters of MBP–*Nm*B-polyST were determined for the donor substrate CMP-Neu5Ac (Fig. 10B, Table 1). The calculated *K*_m_ value of 0.42 mM is fivefold higher than that obtained for the membrane bound enzyme from *E. coli* K1 polyST (Kundig *et al*., 1971; Vijay and Troy, 1975). However, because apart from enzyme source and preparation but also assay and buffer conditions differ between earlier and current studies, further interpretation of the observed variations in *K*_m_ appears unreasonable. In summary, the enzyme-linked assay provided a useful tool to carry out kinetic analysis of polySTs and greatly facilitated the acquisition of data which, until now, have required single-point measurements and elaborate detection methods.

**Table 1:** Kinetic parameters of wild-type and mutant *Nm*B-polyST.

	CMP-Neu5Ac^a^		Colominic acid^b^
		
*Nm*B-polyST	*V*_max_ (μmol min^−1^ mg^−1^)	*K*_m_ (mM)	*K*_m_^c^ (mM)
Wild type	25.8 ± 3.6	0.42 ± 0.03	0.63 ± 0.10
H278A	4.0 ± 1.1	1.37 ± 0.12	0.40 ± 0.14
P279A	6.0 ± 1.0	2.15 ± 0.51	0.51 ± 0.13

a Determined at constant acceptor concentration of 0.28 mg ml^−1^ colominic acid.

b Determined at constant donor concentration of 1 mM CMP-Neu5Ac.

c Kinetic constants were calculated based on an average chain length of 32 residues for colominic acid as estimated using the thiobarbituric acid assay procedure according toSkoza and Mohos (1976).

Kinetic values were obtained using the continuous polysialyltransferase assay and are presented as the means ± SE of three independent determinations.

### The HP motif is involved in CMP-Neu5Ac binding

**Fig. 11:**
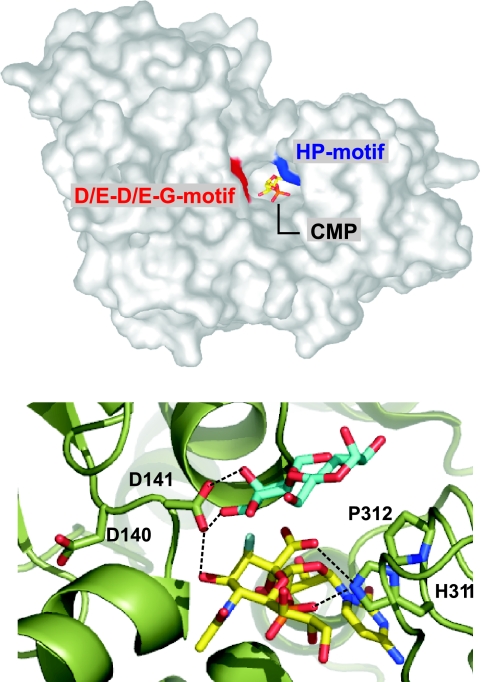
Location of the conserved HP and D/E-D/E-G motifs in the *P. multocida* sialyltransferase *Pm*ST1. A. Surface representation of *Pm*ST1 (PDB entry 2EX1). The motifs are coloured in red (D/E-D/E-G) and blue (HP) while bound CMP is shown in yellow stick representation. B. Active site view of *Pm*ST1. Amino acids that are part of the two motifs are labelled and depicted in green stick representation. Hydrogen bonds are shown as dotted line while the bound donor analogue CMP-3F(α)-Neu5Ac is depicted in yellow and the bound acceptor lactose is shown in cyan. Figures were generated with Pymol (http://www.pymol.org).

Because *Nm*B-polySTs carrying the point mutations H278A and P279A retained residual activity *in vitro*, kinetic properties of these mutants could be determined. Both mutants were expressed as MBP fusion proteins, purified and tested in the enzyme-linked polyST assay. As listed in Table 1, *V*_max_ values for CMP-Neu5Ac were decreased by a factor of 4 (P279A) and 6 (H278A) with respect to the wild-type enzyme and, in both proteins, *K*_m_ values for CMP-Neu5Ac were increased three- to fivefold. These data suggest that the HP motif of *Nm*B-polyST is involved in binding of the donor substrate CMP-Neu5Ac. To further analyse the effect of both mutations on acceptor binding, *K*_m_ values for colominic acid were determined. Interestingly, Michaelis constants were not significantly influenced by either mutation indicating that: (i) the HP motif does not vitally participate in acceptor binding, and (ii) the introduced point mutations did not cause major structural changes as the acceptor binding site appeared to be largely unaffected. Remarkably, the recently solved crystal structure of *P. multocida* sialyltransferase *Pm*ST1 (Ni *et al*., 2006) revealed a similar CMP-Neu5Ac binding function of the HP motif in the GT-80 sialyltransferase family. The enzyme consists of two Rossmann domains that form a deep cleft in which the active site is located and has been crystallized in the presence and absence of donor and acceptor substrates. The active site of *Pm*ST1 with bound donor analogue CMP-3F(α)Neu5Ac and acceptor lactose (Ni *et al*., 2007) is shown in Fig. 11 and illustrates that the histidine residue of the HP motif (H311 in *Pm*ST1) is directly involved in CMP-Neu5Ac binding. It forms hydrogen bonds with the phosphate group of CMP and with the carboxylate function of the sialic acid moiety. Moreover, this structure shows that also the D/E-D/E-G motif is located directly at the active site cleft of *Pm*ST1 (DDG) and is involved in binding of donor and acceptor substrates. The second aspartic acid residue of the DDG sequence forms hydrogen bonds to the acceptor lactose and to the hydroxyl group in position C4 of the sialic acid moiety. In combination, the structural and biochemical data provide strong evidence that the D/E-D/E-G and HP sequences are crucial for CMP-Neu5Ac binding and enzyme catalysis, in bacterial sialyl- and polysialyltransferases that harbour these motifs.

## Discussion

In this study, we functionally characterized the polysialyltransferase responsible for capsule biosynthesis in *N. meningitidis* serogroup B. Although polySTs are interesting therapeutic targets to combat these pathogens, no data on isolated proteins and virtually no information on structure–function relationships, which are crucial for the rational design of inhibitors, have been reported. Attempts to purify or solubilize membrane-associated polySTs have failed or resulted in inactivation of the enzymes, so that membrane association was proposed to be essential for polyST activity (Steenbergen and Vimr, 2003; Vionnet *et al*., 2006).

With the aim of overcoming the lack in structure–function information, we started this study by screening for production systems that enable expression and purification of active *Nm*B-polyST. Soluble expression of active protein was significantly improved after addition of large N-terminal fusion parts and allowed purification of *Nm*B-polyST fused to MBP. Purified MBP–*Nm*B-polyST was enzymatically active and, as described for membrane bound bacterial polySTs of *E. coli* K1 and K92 (Steenbergen and Vimr, 1990; Ferrero *et al*., 1991; Chao *et al*., 1999), able to synthesize long polySia chains from oligomeric primers of at least DP3. Consequently, our data provide clear evidence that membrane association is not a prerequisite for *Nm*B-polyST activity. Furthermore, we demonstrate that the purified enzyme elongates polySia chains in a non-processive manner. This is in contrast to *in vivo* studies carried out in *E. coli* K1 that suggest a processive mode of polySia biosynthesis (Steenbergen and Vimr, 2003). PolySTs were proposed to be part of a large capsule biosynthesis complex, in which biosynthesis and translocation of polySia across the inner and outer bacterial membranes are tightly linked (Steenbergen and Vimr, 2003) and the functional *E. coli* K92 polysialyltransferase complex was found to be larger than a monomer (Vionnet *et al*., 2006). Thus, potential interaction partners of polyST could increase the efficiency of polySia biosynthesis and thereby increase processivity. We (W.F.V. and J.V.) recently demonstrated that also membrane-bound *E. coli* K92 polyST is a non-processive enzyme *in vitro* (Vionnet and Vann, 2007), but the utilized membranes lacked other gene products of the K92 capsule biosynthesis cluster as well. However, even polySia biosynthesis assayed with intact membrane preparations of *E. coli* K1, which most likely include all relevant interaction partners of polyST, was found to be less efficient with exogenously added sialyloligomers than with endogenous or lipid bound acceptors (Cho and Troy, 1994; Chao *et al*., 1999; McGowen *et al*., 2001). This may argue for a more effective binding of the endogenous acceptor and hence result in increased processivity. In conclusion, the finding that recombinant soluble *Nm*B-polyST is non-processive does not exclude processivity of the enzyme in the living system. The investigation of functional properties of recombinant *Nm*B-polyST in complex with other factors of the capsular biosynthetic machinery is therefore an important aim in future studies. Interestingly in this regard, MBP–*Nm*B-polyST expressed in a polyST-deficient *Neisseria* strain is capable to complement the defect (see Fig. 3). The fusion protein may thus provide an interesting tool for studies aimed at identifying polyST-mediated protein–protein interactions *in vivo.*

Mapping of the minimal catalytic domain of *Nm*B-polyST has shown that the C-terminal domain, though not conserved in the homologous *E. coli* enzymes, is essential for catalytic activity. All C-terminally truncated *Nm*B-polySTs were completely inactive. Studies are underway to analyse if potential variations in folding, in the oligomerization status or in substrate binding properties are responsible for inactivation of the truncated polySTs (Fig. 2).

To characterize the catalytic domain of *Nm*B-polyST in more detail, we aimed at identifying key residues for polyST function. So far no functional residues have been described for bacterial polysialyltransferases and no sequence relationships to other bacterial sialyltransferases have been reported. In contrast to the eukaryotic sialyltransferases, which are grouped into a single CAZy family (GT-29) and harbour four highly conserved and well-described sialylmotifs (Drickamer, 1993; Livingston and Paulson, 1993; Geremia *et al*., 1997; Jeanneau *et al*., 2004), the less homologous bacterial sialyltransferases are found in four different CAZy families. GT-38 contains bacterial polysialyltransferases, while GT-42, GT-52 and GT-80 include bacterial LOS sialyltransferases. Heretofore, no conserved sequence features relating members of different CAZy families have been described. In the current study, we combined sequence alignments and visual inspections and identified two short motifs in bacterial sialyltransferases, the D/E-D/E-G motif and the HP motif. Both motifs are conserved throughout the CAZy families GT-38 and GT-52 and GT-80, with the only exception of a *H. ducreyi* sialyltransferase (AAP9506, HA instead of the HP), and are in addition found in pfam family 05855. The latter contains bacterial sialyltransferases similar to the Lst of *H. ducreyi* that have not yet been included in the CAZy database. Our mutagenesis studies of *Nm*B-polyST demonstrate that the D/E-D/E-G motif is essential for polyST activity. Single-amino-acid substitutions within this motif (E153A and G154A) completely abolished enzyme activity *in vitro* and *in vivo*. In contrast, the simultaneous mutation of H278 and P279 of the HP motif to alanine is required to destroy polyST activity. Single mutations within this motif did not obstruct polyST activity but resulted in reduced capsule production and lowered catalytic efficiency of the enzyme as shown by *in vivo* studies and kinetic analysis of the purified mutants. Most interestingly, the HP motif appears to be involved in binding of the donor substrate as reflected by increased *K*_m_ values for CMP-Neu5Ac in the H278A and P279A mutants. In contrast, binding of the acceptor was not influenced. The determined Michaelis constants for colominic acid were not significantly affected which furthermore argues against severe misfolding of these mutants.

Support for the functional relevance of the D/E-D/E-G and the HP motif in bacterial sialyltransferases is furthermore provided by the recently solved crystal structure of the *P. multocida* sialyltransferase *Pm*ST1 (Ni *et al*., 2006). *Pm*ST1 is a member of CAZy family GT-80 and belongs to the glycosyltransferase-B structural superfamily. Interestingly, this fold was also predicted for the bacterial polysialyltransferases of GT-38 including *Nm*B-polyST (Breton *et al*., 2006). *Pm*ST1 harbours both motifs (D/E-D/E-G and HP) close to its active site cleft, whereby the histidine residue of the HP motif (H311) forms a hydrogen-bond to the phosphate group of bound CMP. Mutation of this residue to alanine resulted in a 30-fold reduction of activity and a twofold increase in *K*_m_ for CMP-Neu5Ac and it was suggested that H311 stabilizes the CMP leaving group in *Pm*ST1 catalysis (Ni *et al*., 2007). As the H278A mutation had similar effects on the kinetic properties of *Nm*B-polyST, this residue may have a related function in polyST catalysis. So far no data on mutagenesis of the equivalent proline residue (P312) of *Pm*ST1 are available. However, mutation of this residue to alanine is likely to influence the structure of the loop harbouring the HP motif and may thereby displace the histidine residue. A similar effect may explain the reduced activity of the P279A mutation in *Nm*B-polyST. Strikingly, residues of the D/E-D/E-G motif were also found to be essential for *Pm*ST1 function. Although the first aspartic acid residue (D140) of the motif points towards the protein core and is most likely not involved in substrate interactions, the second aspartic acid (D141) protrudes into the active site cleft of *Pm*ST1 (Fig. 11). Moreover, this residue was shown to interact with the *Pm*ST1 acceptor lactose and suggested to act as general base in *Pm*ST1 catalysis. Mutation of D141 to alanine virtually inactivated the enzyme (20 000-fold reduction of activity) (Ni *et al*., 2007). This is again in perfect agreement with the inactive E153A mutant of *Nm*B-polyST and may suggest an analogous function of this residue in polyST catalysis. Indirect evidence that the protein stretch containing the conserved D/E-D/E-G motif is located close to the active site also in CAZy GT-52 family members was provided by Wakarchuk *et al*. (2001). They showed that the neisserial α2,3-sialyltransferase Lst switches to a bifunctional α2,3/6-sialyltransferase mode upon mutation of the single-residue G168 and predicted this residue to be positioned in an acceptor-binding cavity. Interestingly, G168 is found only two residues upstream of the D/E-D/E-G motif (residues 164–166) in this enzyme. Finally it should be mentioned that structural information is available for a second sialyltransferase the cst-II from *C. jejuni* (Chiu *et al*., 2004). However, this enzyme is a member of CAZy family GT-42, which is the only bacterial sialyltransferase family so far that does not harbour HP- and D/E-D/E-G motifs.

In conclusion, the establishment of efficient expression, purification and assay procedures for *Nm*B-polyST allowed us to identify and characterize key groups for polyST function. Alignments with bacterial sialyltransferases revealed two functional motifs, the D/E-D/E-G and the HP motif, that are conserved in otherwise unrelated bacterial sialyltransferases of CAZy families GT-38, GT-52 and GT-80 as well as in pfam 05855. The functional importance of both motifs for enzyme catalysis and/or CMP-Neu5Ac binding was demonstrated by mutational analysis of *Nm*B-polyST and is emphasized by structural and biochemical data available for the *P. multocida* sialyltransferase *Pm*ST1. Our data therefore allow hypothesizing that basic features of substrate binding and enzyme catalysis are conserved in a wide range of bacterial sialyltransferases and improve the basis for design of sialyltransferase-specific drugs.

## Experimental procedures

### Materials

Colominic acid and CMP-Neu5Ac were purchased from Sigma. Oligomeric sialic acids were from Nacalai Tesque. The pMAL-c Vector was from New England Biolabs, and pET vectors were purchased from Novagen.

### Cloning of *Nm*B-polyST expression vectors

*Nm*B-polyST was amplified by PCR using plasmid pUE3 (Frosch *et al*., 1991) as template and the primer pair KS23 (5′-G CAT GGA TCC CTA AAG AAA ATA AAA AAA GCT-3′) and KS41 (5′-GCA GGC GGC CGC TCT ATC TCT ACC AAT TCT-3′) containing BamHI and NotI sites (underlined) respectively. The PCR product was subcloned into the respective sites of pET23a to result in an N-terminally T7- and C-terminally His_6_-tagged construct (pET23a-*Nm*B-polyST). To generate an expression construct including an N-terminal NusA fusion part followed by a StrepII-tag, adapters Strepa (5′-CTA GTG CTA GCT GGA GCC ACC CGC AGT TCG AAA AAG GCG CCC TGG TTC CGC GTG-3′) and Strepas (5′-GAT CCA CGC GGA ACC AGG GCG CCT TTT TCG AAC TGC GGG TGG CTC CAG CTA GCA-3′) were inserted by adapterligation into the BamHI and SpeI restriction sites of pET43a generating pET43a-Strep. The *Nm*B-polyST gene was subcloned from pET23a-*Nm*B-polyST using restriction sites BamHI and NotI to generate the expression vector pET43a-Strep-*Nm*B-polyST that encodes for the polyST fused with an N-terminal NusA/Strep-tag and a C-terminal His_6_-tag. To obtain the vector pMBP-Strep, the MBP was amplified by PCR with primers FF03 (5′-GAT ATT CAT ATG AAA ACT GAA GAA GGT AAA CT-3′) and FF04 (5′-CAT ATA CTA GTC CTA CCC TCG ATG GAT CC-3′) from the vector pMAL-c and subsequently subcloned into the NdeI and SpeI restriction sites of pET43a-Strep. Finally, *Nm*B-polyST was subcloned from pET43a-Strep-*Nm*B-polyST using the NheI/NotI sites resulting in pMBP-Strep-*Nm*B-polyST that encodes for the polyST fused with an N-terminal MBP/Strep-tag and a C-terminal His_6_-tag.

### Generation of truncated proteins

N-terminally truncated *Nm*B-polySTs were generated by PCR using the forward primers IO07 (5′-GCA TGG ATC CAC ATC TCC ATT TTA TCT TAC-3′) for Δ23 *Nm*B-polyST, IO08 (5′-GCA TGG ATC CAA CAA TTT ATT TGT CAT ATC TA-3′) for Δ33 *Nm*B-polyST and IO09 (5′-GCA TGG ATC CTT ATA TAC TTC TAA AAA CTT AAA A-3′) for Δ64 *Nm*B-polyST and the reverse primer KS41 (5′-GCA GGC GGC CGC TCT ATC TCT ACC AAT TCT-3′). C-terminally truncated *Nm*B-polySTs were generated by PCR using the reverse primers FF01 (5′-GCA TGC GGC CGC ATC TTT ACT ATG AAA GTC-3′) for *Nm*B-polyST Δ22, FF02 (5′-GCA TGC GGC CGC CCC TAA TAA GGT AAT ATT G-3′) for *Nm*B-polyST Δ60, KS335 (5′-G CAG GCG GCC GC TTC AAA TGT TTC TTC TGT TTT AAA-3′) for *Nm*B-polyST Δ94, KS334 (5′-GC AG GCG GCC GC AAA TGT TTC TTC TGT TTT AAA GA-3′) for *Nm*B-polyST Δ95 and FF02 (5′-GCA GGC GGC CGC TTC TTC TGT TTT AAA GAG AG-3′) for *Nm*B-polyST Δ97 and the forward primer KS333 (5′-G CAT GGA TCC CTA AAG AAA ATA AAA AAA GCT CTT-3′). BamHI and NotI sites (underlined) in forward and reverse primers, respectively, were used for subcloning of the PCR products into the BamHI/NotI sites of pET43a-Strep. The resulting constructs encode for proteins with an N-terminal NusA-/Strep-tag and a C-terminal His_6_-tag. The identity of all constructs was confirmed by sequencing.

### Site-directed mutagenesis

Single-point mutations of *Nm*B-polyST were obtained by QuickChange site-directed mutagenesis (Stratagene) following the manufacturer's instructions using the plasmid pET23a-*Nm*B-polyST as template. Mutated polySTs were subsequently subcloned into the BamHI and NotI sites of pET23a resulting in expression of N-terminally T7-tagged proteins. The identity of all constructs was confirmed by sequencing. Mutagenic primers are given below with the mutated base triplets underlined: pET23a-G154A: KS52 (5′-G ACT CAT TTA ATT GAT GAA GCG ACT GGA ACA TAT GCT CC-3′) and KS53 (5′-GG AGC ATA TGT TCC AGT CGC TTC ATC AAT TAA ATG AGT C-3′); pET23a-E153A: KS54 (5′-T ACG ACT CAT TTA AT T GAT GCA GGG ACT GGA ACA TAT GC-3′) and KS55 (5′-GC ATA TGT TCC AGT CCC TGC ATC AAT TAA ATG AGT CGT A-3′); pET23a-H278A: KS56 (5′-ATT AAA GGA AAG ATA TTT ATT AAA CTA GCC CCA AAA GAG ATG GGC AAC AAC-3′) and KS57 (5′-GTT GTT GCC CAT CTC TTT TGG GGC TAG TTT AAT AAA TAT CTT TCC TTT AAT-3′); pET23a-P279A: KS58 (5′-GGA AAG ATA TTT ATT AAA CTA CAC GCA AAA GAG ATG GGC AAC AAC TA-3′) and KS59 (5′-TA GTT GTT GCC CAT CTC TTT TGC GTG TAG TTT AAT AAA TAT CTT TCC-3′). Mutants H278A and P279A were additionally introduced into the pMBP-Strep vector. Therefore, the polyST inserts of pET23a-H278A and pET23a-P279A were ligated into pET43a-Strep using restriction sites BamHI and NotI and subsequently subcloned into pMBP-Strep using the NheI and NotI sites.

### Expression of recombinant *Nm*B-PolyST

To optimize protein production, bacteria were either cultivated at 30°C (only polySTs fused to NusA) or at 15°C. For production at 30°C, freshly transformed *E. coli* BL21(DE3) were grown in PowerBroth medium (Athena ES) containing 200 μg l^−1^ carbenicillin at 30°C and 225 r.p.m. At an optical density of OD_600_ = 1.8 expression was induced by adding 1 mM IPTG. Bacteria were harvested 3 h after induction by centrifugation (6000 *g* for 15 min, 4°C). For 15°C production, bacteria were grown at 30°C to an optical density of OD_600_ = 0.9. Cultures were then rapidly cooled (ice bath) and further grown at 15°C until OD_600_ = 1.8 was reached. Transgene expression was induced by the addition of 1 mM IPTG and cells were harvested 24 h after induction. All pellets were washed once with PBS and stored at −20°C.

### Separation of soluble and insoluble fractions of *Nm*B-PolyST

To analyse *Nm*B-PolyST expression and enzymatic activity in bacterial lysates, cells were re-suspended in 50 mM Tris-HCl pH 8.0, 40 mM MgCl_2_ and lysed by sonication. Soluble and insoluble fractions were obtained following centrifugation (16 000 *g*, 15 min, 4°C). The insoluble fraction (pellet) was re-suspended in 50 mM Tris-HCl pH 8.0, 40 mM MgCl_2_ in a volume equal to that of the soluble fraction.

### Purification of recombinant MBP–*Nm*B-PST fusion protein

Bacterial pellets from 0.5 l of cultures were re-suspended in binding buffer (20 mM Tris; pH 7.4; 1 mM EDTA; 1 mM DTT; 25 mM NaCl) including protease inhibitors (40 mg ml^−1^ Bestatin, 1 μg ml^−1^ Pepstatin and 1 mM PMSF) to give a final volume of 20 ml. Cells were disrupted by sonication and samples were centrifuged (30 min; 16 000 *g*, 4°C) and filtered (Sartorius Minisart 0.8 μm). For affinity absorption of MBP–*Nm*B-polyST, pre-swollen amylose resin (New England Biolabs) was added to the cleared supernatant and incubated at 4°C for 1 h. Subsequently, the incubation mixture was transferred to a column and washed with 12 volumes of binding buffer at a flow rate of 0.5 ml min^−1^. Bound protein was eluted with elution buffer (binding buffer containing 10 mM maltose). Fractions containing the fusion protein were pooled and passed through a desalting column (High Prep 26/10) equilibrated in 50 mM NaH_2_PO_4_ pH 8.0 and finally concentrated to 2 mg ml^−1^ using Amicon Ultra centrifugal devices (Millipore). To further enrich the MBP–*Nm*B-polyST fusion protein, a gel filtration chromatography step was applied. Samples were filtered (Millipore Ultrafree MC 0.2 μm) and loaded on a Superdex 200 10/300 GL column (GE Healthcare). Proteins were eluted at a flow rate of 0.5 ml min^−1^ with 50 mM NaH_2_PO_4_ pH 8.0 buffer and fractions of 0.5 ml were collected. Obtained protein samples were stable for more than 3 weeks if stored at 4°C.

### SDS-PAGE and immunoblotting

SDS-PAGE was performed under reducing conditions using 2.5% (v/v) β-mercaptoethanol and 1.5% (w/v) SDS. For Western blot analysis, proteins were blotted onto nitrocellulose (Whatman). Proteins containing an N-terminal StrepII-tag were detected by StrepTactin-alkaline phosphatase-conjugate (StrepTactin-AP; IBA) according to the manufacturer's guidelines. His-tagged proteins were detected with 1 μg ml^−1^ penta-His antibody (Qiagen) followed by goat anti-mouse IgG-AP (Dianova). For quantification by infrared fluorescence detection, samples and standard proteins were blotted onto PVDF membranes (Millipore). His-tagged proteins were detected with 1 μg ml^−1^ penta-His antibody (Qiagen) followed by 50 ng ml^−1^ goat anti-mouse IR680 antibody (LI-COR) and quantified according to the recommendations of the Odyssey infrared imaging system (LI-COR).

### Radiochemical polyST assays

PolyST activity in bacterial lysates was analysed as described previously (Weisgerber *et al*., 1991). Briefly, 10 μl of lysate was mixed with 10 μl of TMD buffer (40 mM MgCl_2_, 50 mM, Tris/HCl pH 8.0) and 2 μl of colominic acid (100 mg ml^−1^) as acceptor and reactions were started by adding 2 μl of CMP-[^14^C]-Neu5Ac (13 mM, 1.55 mCi mmol^−1^). Samples were incubated at 37°C and 5 μl of aliquots were spotted on Whatman 3MM CHR paper after the respective reaction time. Following descending paper chromatography, the chromatographically immobile ^14^C-labelled polyST reaction products were quantified by scintillation counting. To analyse the products of purified *NmB*-polyST in more detail, reaction mixtures were analysed in the TBE-buffered (90 mM Tris, 90 mM borate, 2 mM EDTA, pH 8.3) electrophoresis system described for analysis of acidic capsular polysaccharides (Pelkonen *et al*., 1988). Enzyme reactions were carried out in TMD in a total volume of 24 μl as described above, using 10 μg of enzyme and 0.001–1 mM α2,8-linked sialic acid (DP5) as acceptor. Control samples were subsequently treated with 1 μg of polySia-degrading endoN (Stummeyer *et al*., 2005) for 20 min at 37°C. Equal volumes of sample buffer (2 M sucrose in TBE) were added and samples were electrophoresed at 4°C and 200 V overnight. To visualize ^14^C-labelled reaction products, gels were vacuum-dried immediately after electrophoresis and exposed to an imaging film (BioMax, Kodak).

### Fluorescent polyST assays

PolyST activity of purified *Nm*B-polyST was also monitored using the fluorescent acceptor GT3-FCHASE as acceptor (Vionnet and Vann, 2007). Briefly, GT3-FCHASE (0.23 μM), CMP-Neu5Ac (50–500 mM) and purified *Nm*B-polyST (30*–*180 μg ml^−1^) were incubated in TMD buffer (40 mM MgCl_2,_ 50 mM, Tris/HCl pH 8.0) at 37°C. At the indicated time points (2–30 min) reactions were stopped by adjusting to 25% ethanol and samples were further analysed by HPLC as described (Vionnet and Vann, 2007).

### Continuous spectrophotometric polysialyltransferase assay

For rapid characterization of purified polysialyltransferases, the glycosyltransferase testing system described by Gosselin *et al*. (1994) was adapted to polysialyltransferases. All measurements were carried out in 96-half area well plates (Greiner Bio-one) in a total volume of 106.5 μl. In detail, a master solution was prepared containing the linking enzymes pyruvate kinase (16.5 U ml^−1^, Sigma), lactate dehydrogenase (23.5 U ml^−1^, Sigma) and nucleotide monophosphate kinase (0.5 U ml^−1^, Roche) in reaction buffer [1.5 mM ATP (Sigma), 1 mM PEP (Fluka), 0.13 mM NADH (Roche), 14 mM MgSO_4_, 56 mM KCl in 100 mM Tris pH 7.5]. As acceptor substrate either colominic acid or sialyloligomers were added in various concentrations. After addition of the donor substrate CMP-Neu5Ac (62 μM to 2 mM) samples were first monitored at OD 340 nm until a stable baseline was reached (3–5 min). This was essential to metabolize free CMP, which is always present in CMP-Neu5Ac preparations due to hydrolysis of the substrate. Finally, PST was added (10–20 μg ml^−1^) and reactions were followed until the total amount of NADH was metabolized.

### Generation and analysis of mutant *Neisseria* strains

For mutagenesis of *N. meningitidis* strains, the plasmids pET23a-G154A, pET23a-E153A, pET23a-H278A and pET23a-P279A needed to be modified to allow regular homologous recombination into the meningococcal chromosome and selection for the mutants. The *siaD* downstream region was amplified from serogroup B strain MC58 with primers GH149 (5′-GCG CGC CTC GAG AAT ACT ATG ACT TCT GA TCT CC-3′) and GH150 (5′-GCG CGC CTC GAG CGA GTA ATT TGA CAA TAG AGC G-3′) and integrated into each plasmid downstream of the mutated *siaD* gene using the respective XhoI sites (underlined). The resulting plasmids were linearized with NotI, blunt ended with T4 DNA polymerase and ligated with the kanamycin resistance cassette excised from pUC4K (GE Healthcare) by HincII. The final plasmids harbouring the kanamycin resistance cassette between the *siaD* gene and the *siaD* downstream region were used to transform serogroup B meningococcal strain MC58. Transformants were selected on GC agar supplemented with 100 μg ml^−1^ kanamycin. Recombination of the mutagenized motifs into the meningococcal *siaD* gene was verified by sequencing the PCR product obtained with primer pair GH157 (5′-CA GGC CAC TAC TCC TAT C TG-3′)/Kana2 (5′-GAT TTT GAG ACA CAA CGT GG-3′) with either primers GH157 and GH160 (5′-AGG TTC ATT AAT AAC TAC CAG C-3′) (D/E-D/E-G motif) or primers UE8a (5′-AA CGC TAC CCC ATT TCA-3′) and GH160 (HP motif). Furthermore, regular homologous recombination was confirmed by Southern blot hybridizations with the *siaD* gene and the kanamycin resistance gene used as a probe respectively. The meningococcal capsule phenotype was analysed with mab 735 by slide agglutination as described previously (Vogel *et al*., 2001). Quantitative analysis of capsule expression was performed by whole-cell ELISA as described (Vogel *et al*., 2001). Briefly, microtitre plates were pre-coated with poly d-lysine (25 μg ml^−1^ in PBS) for 1 h at room temperature. After three washing steps with PBS, bacterial suspensions (20 μl well^−1^, OD_600_ = 0.10 in PBS) were applied for 2 h and cross-linked to poly d-lysine by adding glutaraldehyde (0.05% in PBS) for 10 min. Plates were washed three times with PBS and non-specific binding sites were saturated by incubation with 1% BSA in PBS for 1 h. After three washing steps capsular polySia was detected by immunostaining using the polySia-specific mab 735. The amount of bacteria bound to each well was controlled with a parallel set of microplates using mab P1.7 directed against the PorA antigen of the meningococcal strain MC58 for detection. Subsequently, plates were incubated with peroxidase coupled secondary antibody (Dianova) and analysed by colour reaction.

### *In vivo* analysis of MBP–*Nm*B-polyST

Neisserial expression constructs were generated for *in vivo* comparison of wild type and MBP–*Nm*B-polyST. The MBP–polyST insert of the *E. coli* expression construct pMBP-Strep-*Nm*B-polyST was amplified with primers HC574 (5′-GCG CGC TCT AGA GAA GGA GAT ATA CAT ATG AAA AC-3′) and HC572 (5′-GCG CGC GAT ATC TTA GTG GTG GTG GTG GTG G-3′) and integrated between the SpeI and EcoRV sites of the neisserial expression vector pAP1 (Lappann *et al*., 2006) resulting in pAP1-MBP–*Nm*B-polyST. For comparison also the polyST gene without further tags was amplified with primers HC573 (5′-GCG CGC GAT ATC AGA GAT ACA ATA ATG CTA AAG AAA ATA AAA AAA GC-3′) and HC572 and integrated into the EcoRV site of pAP1 resulting in pAP1-*Nm*B-polyST. Further *in vivo* studies were performed using strain 2517, an unencapsulated polyST knockout mutant of the meningococcal serogroup C strain 2120 (Ram *et al*., 2003) that was transformed with the resulting plasmids. Wild type and transformants were analysed for capsule expression by whole-cell ELISA as described above. Bacterial loading was controlled using anti-PorA antibody P1.2. Additionally, neisserial lysates were analysed by Western blot analysis. Bacteria were pelleted from 2 ml of suspension cultures (OD_600_ = 1.1), re-suspended in 100 μl of Lämmli sample buffer [100 mM Tris pH 6.8, 1.7% SDS, 16.5% glycerol (v/v), 2.5% 2-mercaptoethanol, bromophenol blue] and lysed by sonication. The lysates were centrifuged (16.000 *g*, 10 min, 4°C) and supernatants were divided in two aliquots. One aliquot was subsequently digested with 1 μg of endoN (Stummeyer *et al*., 2005) and incubated for 15 min at room temperature followed by a 15 min incubation at 37°C. All samples were incubated for 10 min at 60°C prior to electrophoresis. SDS-PAGE and Western blot were performed as described above.

## Supplementary material

The following supplementary material is available for this article:

Fig. S1 Elongation of trimeric a-2,8-linked sialic acid (DP3) by purified NmB-polyST.

This material is available as part of the online article from: http://www.blackwell-synergy.com/doi/abs/10.1111/j.1365-2958.2007.05862.x (This link will take you to the article abstract).
