# Supplementary material for: Biochemical characterization of a Neisseria meningitidis polysialyltransferase reveals novel functional motifs in bacterial sialyltransferases
Source: Mol Microbiol. 2007 Sep;65(5):1258–75. doi: 10.1111/j.1365-2958.2007.05862.x (PMC2169525; doi:10.1111/j.1365-2958.2007.05862.x)
Supplement: Fig. S1 — Elongation of trimeric a-2,8-linked sialic acid (DP3) by purified NmB-polyST. [file mmi0065-1258-SD1.pdf]

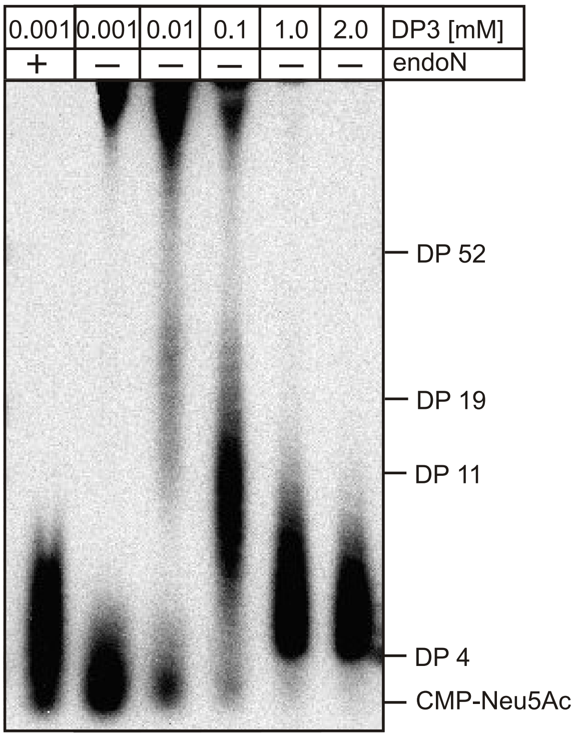

Figure S1. *Elongation of trimeric α-2,8-linked sialic acid (DP3) by purified NmB-polyST.* (A) Purified *Nm*B-polyST was assayed for 30 min in the presence of 1 mM CMP-[C14]Neu5Ac and increasing concentrations of DP3 as indicated. The sample including 0.001 mM DP3 was prepared as duplicate to subsequently digest the extra probe with polySia-specific endoN. Radiolabelled reaction products were separated by acrylamide-gelelectrophoresis (25 %) and detected by autoradiography. The following dyes were used as standards and correspond to polySia chain length given in brackets: trypan blue (DP100), xylene cyanol (DP52) bromophenol blue (DP19), bromcresol purple (DP11), phenole red (DP4).
